# A Trinocular System for Pedestrian Localization by Combining Template Matching with Geometric Constraint Optimization

**DOI:** 10.3390/s25195970

**Published:** 2025-09-25

**Authors:** Jinjing Zhao, Sen Huang, Yancheng Li, Jingjing Xu, Shengyong Xu

**Affiliations:** 1 School of Electronics, Peking University, Beijing 100871, China; zhaojinjing@pku.edu.cn (J.Z.); senhuang@stu.pku.edu.cn (S.H.); lycheng@pku.edu.cn (Y.L.); 2 School of Integrated Circuits, Shandong University, Jinan 250100, China; xujj@sdu.edu.cn

**Keywords:** trinocular vision, pedestrian localization, template matching

## Abstract

Pedestrian localization is a fundamental sensing task for intelligent outdoor systems. To overcome the limitations of accuracy and efficiency in conventional binocular approaches, this study introduces a trinocular stereo vision framework that integrates template matching with geometric constraint optimization. The system employs a trinocular camera configuration arranged in an equilateral triangle, which enables complementary perspectives beyond a standard horizontal baseline. Based on this setup, an initial depth estimate is obtained through multi-scale template matching on the primary binocular pair. The additional vertical viewpoint is then incorporated by enforcing three-view geometric consistency, yielding refined and more reliable depth estimates. We evaluate the method on a custom outdoor trinocular dataset. Experimental results demonstrate that the proposed approach achieves a mean absolute error of 0.435 m with an average processing time of 3.13 ms per target. This performance surpasses both the binocular Semi-Global Block Matching (0.536 m) and RAFT-Stereo (0.623 m for the standard model and 0.621 m for the real-time model without fine-tuning). When combined with the YOLOv8-s detector, the system can localize pedestrians in 7.52 ms per frame, maintaining real-time operation (>30 Hz) for up to nine individuals, with a total end-to-end latency of approximately 32.56 ms.

## 1. Introduction

Reliable pedestrian localization is a fundamental requirement in a wide range of domains, including autonomous driving [1], service robotics [2,3], and assistive navigation [4,5]. In urban sidewalk environments, pedestrians move dynamically among buildings, street infrastructure, and other mobile agents. Even minor localization errors in such safety-critical contexts can have severe consequences, whether for autonomous vehicles maneuvering safely in traffic [6] or wearable assistive devices guiding visually impaired users [7]. Therefore, stereo vision has been widely adopted as a perception technology [8,9,10]. By emulating human binocular vision, it provides dense depth cues without external infrastructure, offering a flexible and scalable solution for spatial perception [11,12,13].

Over the past two decades, binocular stereo vision has developed rapidly [14,15]. Early methods, such as Block Matching (BM) [16] and Semi-Global Matching (SGM) [17], established the foundation for efficient disparity estimation. More recently, deep learning approaches, such as GC-Net [18] and PSMNet [19], established the paradigm of deep cost aggregation. Subsequent works, including RAFT-Stereo [20] and its variants [21,22], have substantially improved disparity accuracy and robustness under challenging conditions such as occlusion or low-texture regions. However, inherent limitations remain. The geometric configuration of binocular systems confines estimation to a single baseline plane, resulting in depth uncertainty that increases quadratically with distance [23]. This limitation severely compromises long-range pedestrian localization [24]. Moreover, deep learning–based solutions, while powerful, often impose high computational costs that hinder deployment in lightweight, real-time systems [25,26,27]. These challenges underscore the need for stereo vision methods that are both more robust and computationally efficient, particularly in outdoor pedestrian localization.

A common strategy for pedestrian localization is to combine object detection networks with depth information derived from stereo vision systems [28,29,30]. For instance, Zuo et al. [30] utilize the Semi-Global Block Matching (SGBM) algorithm to acquire a depth matrix of the platform scene and employ the YOLOX object detection algorithm to identify the positions of passengers on subway platforms. Although effective for scene understanding, dense stereo methods are often computationally redundant for target-specific localization tasks, as they devote significant resources to background regions irrelevant to the object of interest. To achieve efficient target positioning, researchers have developed feature-based methods in binocular stereo vision, which involve extracting and matching feature points. These approaches are computationally efficient and have been applied to pedestrian localization [31,32,33]. For example, Wei et al. [32] proposed a remote distance ranging method based on an improved YOLOv5. In another study, Guo et al. [33] developed a positioning system incorporating edge detection and scale-invariant feature transforms. However, these binocular approaches depend on a limited number of feature points within the target region. For distant pedestrians, these features can be sparse or unreliable, leading to noisy depth estimates and frequent mismatches. The trade-off between the computational overhead of dense methods and the instability of sparse methods has motivated research into hybrid strategies. Leveraging compact attention mechanisms has emerged as a promising alternative to processing dense cost volumes, offering a path toward solutions that are both lightweight and accurate. The attention mechanism improves matching accuracy by effectively fusing spatial context with geometric correspondence cues [34,35]. Moreover, adopting a multi-scale architecture can further improve the fidelity of details [36,37]. Despite these algorithmic advances, fundamental geometric limitations of a two-camera system still bring the challenge of depth uncertainty increasing quadratically with distance.

Trinocular stereo vision has recently been proposed as a promising extension to overcome these shortcomings [38,39,40]. By introducing a third viewpoint vertically offset from the horizontal baseline, trinocular systems form a more complete perception cone [41]. This configuration enriches disparity estimation, improves depth accuracy at longer ranges, and increases resilience against occlusions [42,43]. Researchers have utilized trinocular systems for applications such as scene reconstruction and robotic navigation, where the additional view has improved the robustness of visual odometry and enabled more accurate 360-degree 3D reconstruction [44] and enable more accurate 360-degree 3D reconstruction [45]. In the field of metrology, trinocular vision has been successfully applied to specialized measurement tasks, such as determining the ground clearance of transmission lines [46], measuring spatially encoded artifacts [47], and building intelligent object measurement systems [48].

Although trinocular vision holds considerable potential, research in this area remains limited, and few methods directly address the demands of pedestrian localization in outdoor sidewalk environments. One reason is the added algorithmic complexity and computational overhead that come with processing a third viewpoint. Many existing studies emphasize holistic tasks such as dense disparity estimation or large-scale scene reconstruction [42,43,45], which introduce high costs unsuited to real-time pedestrian localization. Others [46,48] treat trinocular vision simply as multiple binocular pairs, overlooking the geometric advantages of the full system. In addition, current trinocular solutions are often designed for specialized metrology applications, relying on well-defined object edges or controlled environments. Consequently, these pipelines do not adapt well to dynamic and unstructured settings, where challenges such as long-range pedestrian localization remain unresolved. Despite the theoretical advantages of trinocular geometry, its practical potential is still largely unrealized, leaving a critical gap in safety-critical applications like pedestrian localization.

In this work, we present a trinocular stereo vision method designed specifically for pedestrian localization in outdoor sidewalk scenes. The proposed system arranges three cameras in an approximately equilateral triangle configuration, which broadens the effective baseline and introduces a vertical viewpoint that contributes complementary disparity information. Building on this geometry, we first employ a multiscale template matching algorithm to rapidly estimate the pedestrian’s position from the primary binocular pair, ensuring efficient computation without sacrificing accuracy. This initial estimate is then refined by exploiting the third vertical viewpoint through an optimization strategy that combines three-view geometric constraints with similarity measures. By uniting these elements, the proposed framework achieves both high depth accuracy and computational efficiency, offering a practical pathway for deploying stereo vision systems in safety-critical pedestrian localization tasks.

This paper is organized as follows: Section 2 details the proposed methodology. Section 3 evaluates the performance of our method on a custom trinocular vision dataset captured on outdoor sidewalks. Section 4 discusses the results and their potential applications. Finally, Section 5 concludes this study.

## 2. Methodology

In this paper, we present a trinocular stereo vision system for pedestrian localization, wherein the cameras are arranged at the vertices of an equilateral triangle with parallel optical axes. We also introduce a trinocular stereo vision method based on local template matching. First, a multi-scale stereo matching algorithm is applied to the left–right pair to establish the initial disparity and corresponding target position. Subsequently, a three-view constraint optimization refines the estimate by incorporating information from all three views.

**Figure 1:**
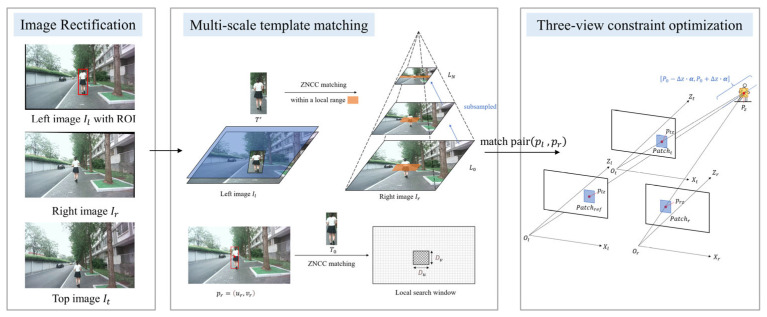
The overall architecture of the proposed trinocular vision-based localization framework. The pipeline begins with rectified trinocular images $I_{l},I_{r},I_{t}$ and an initial region of interest (ROI) in $I_{r}$. Subsequently, multi-scale template matching is performed to obtain an initial depth estimate. Finally, this estimate is refined through a similarity evaluation of image patches that leverages information from all three views to determine a more precise target location.

The overall architecture of the proposed method is illustrated in Figure 1 and the methodology is summarized in Appendix A.

### 2.1. Camera Settings and Symbols

The trinocular camera system is composed of three cameras, designated as left ($C_{l}$), right ($C_{r}$) and top ($C_{t}$), which are arranged in an approximately equilateral triangle. The principal optical axes of the cameras are parallel. The $C_{l}$ and $C_{r}$ cameras constitute the primary binocular stereo pair. The $C_{t}$ is positioned vertically above the midpoint of the $C_{l}-C_{r}$ baseline.

The process of camera calibration and image rectification involves the following steps:

- Intrinsic calibration: Each camera ($C_{l}$, $C_{r}$, $C_{t}$) was calibrated independently to obtain its intrinsic parameters. These parameters include the intrinsic matrix K (which encodes the focal lengths $\left(f_{x},f_{y}\right)$, principal point coordinates $\left(c_{x},c_{y}\right)$ and the vector of distortion coefficients D. The distortion vector accounts for primary radial and tangential lens distortions. The intrinsic parameters are denoted as $\left(K_{l},D_{l}\right)$, $\left(K_{r},D_{r}\right)$ and $\left(K_{t},D_{t}\right)$.
- Extrinsic calibration: The spatial relationship between the cameras was determined by performing extrinsic calibration. With $C_{l}$ serving as the reference coordinate frame, we computed the rotation matrix (R) and translation vector (t) for the $C_{l}$-$C_{r}$ and $C_{l}$-$C_{t}$ pairs, denoted as $\left(R_{lr},t_{lr}\right)$ and $\left(R_{lt},t_{lt}\right)$.
- Image Rectification: The stereo image pair $\left(I_{l},I_{r}\right)$ was rectified using the extrinsic parameters $\left(R_{lr},t_{lr}\right)$ to align epipolar lines, yielding the reprojection matrix $Q_{lr}$, the relative rotation $R_{lr}^{\prime }$, the relative translation $t_{lr}^{\prime }$, and the rotation matrices $R_{rect\_l}$ and $R_{rect\_r}$. Specifically, $R_{rect\_l}$ and $R_{rect\_r}$ represent the transformation from the original camera coordinate systems into their rectified counterparts. The top image $I_{t}$ was undistorted using its intrinsic parameters $\left(K_{t},D_{t}\right)$.

### 2.2. Multi-Scale Template Matching for Initialization

This section introduces an improved multi-scale template matching algorithm for rapid correspondence initialization from a rectified stereo pair $\left(I_{l},I_{r}\right)$. The method employs a two-stage matching strategy to efficiently locate the match. In the first stage, a coarse estimate is established using an image pyramid-based search with a preprocessed template $T^{\prime }$. Subsequently, the second stage refines this estimate by matching the original template $T_{0}$ within a local search window centered on the result from the coarse search.

#### 2.2.1. Template Preprocessing

Prior to the matching process, a template pre-processing step is applied. An original template $T_{0}$ is first acquired from the target pedestrian’s region of interest (ROI) in $I_{l}$, parameterized by its center $\left(u_{l},v_{l}\right)$ and dimensions $\left(w_{0},h_{0}\right)$. The template’s area is calculated as $A_{0}=w_{0}\cdot h_{0}$. To handle variations in scale, a cropped template $T^{\prime }$ with dimensions $\left(w^{\prime },h^{\prime }\right)$ is then defined. The dimensions of $T^{\prime }$ are determined based on the area $A_{0}$ of the original template $T_{0}$, anchored at the same center point $\left(u_{l},v_{l}\right)$, according to the following conditions:

- If $A_{0}<A_{min}$, the template is expanded by a factor $r_{exp}$ to incorporate richer contextual information from the surrounding region. The resulting dimensions are defined as $\left(w^{\prime },h^{\prime }\right)=\left(r_{exp}w_{0},r_{exp}h_{0}\right)$.
- If $A_{0}>A_{max}$, the template is cropped by a factor $r_{cut}$ to remove redundant information, which could degrade matching stability. The resulting dimensions are defined as $\left(w^{\prime },h^{\prime }\right)=\left(r_{cut}w_{0},r_{cut}h_{0}\right)$.
- If $A_{min}<A_{0}<A_{max}$, the template is considered adequate. The resulting dimensions are defined as $\left(w^{\prime },h^{\prime }\right)=\left(w_{0},h_{0}\right)$.

In the following steps, the cropped template $T^{\prime }$ is used for the image pyramid-based search, while the original template $T_{o}$ is used for the precise refinement within the local search window.

#### 2.2.2. Matching Strategy

**Figure 2:**
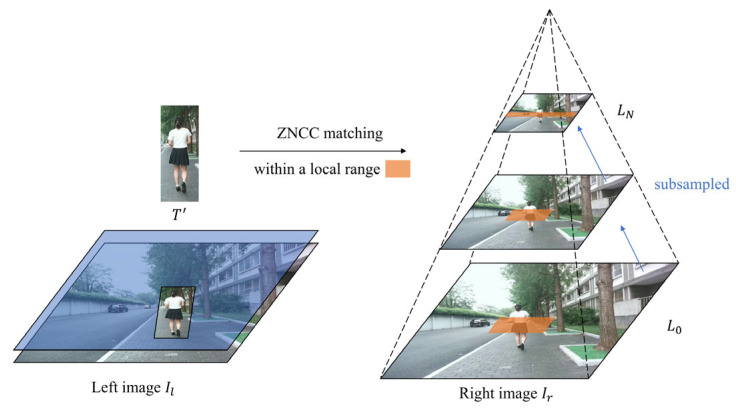
Schematic of the first-stage matching process. The template $T^{\prime }$ is cropped from the left image $I_{l}$. An image pyramid is constructed for the right image $I_{r}$. At each level of the pyramid $L_{k}$, we compute the similarity between the down-sampled template $T_{k}^{\prime }$ and the down-sampled image $I_{r,k}$ within a local search range (highlighted in orange).

To accelerate the search and matching process, we employ an image pyramid strategy. Specifically, we construct an image pyramid for the search image $I_{r}$. Each level of the pyramid is generated by down-sampling the preceding one, as illustrated in Figure 2.

The search proceeds from the top level $L_{N}$ down to the bottom level $L_{0}$. At each level k, the search for the template $T_{k}^{\prime }$ is performed within the down-sampled image $I_{r,k}$. Within the search area of $I_{r,k}$, candidate windows of the same size as $T_{k}^{\prime }$ are extracted. We compute the grayscale image similarity between the template $T_{k}^{\prime }$ and each candidate window $W\left(u,v\right)$ using the zero normalized cross-correlation (ZNCC) score, which is robust to linear and affine illumination changes and thus well-suited for template matching. The function is defined as:

(1) $$ZNCC\left(u,v\right)=\frac{\sum_{i,j}\left(T_{k}^{\prime }\left(i,j\right)-\bar{T_{k}^{\prime }}\right)\cdot \left(W\left(u,v,i,j\right)-\bar{W\left(u,v\right)}\right)}{\sqrt{\sum_{i,j}{\left(T_{k}^{\prime }\left(i,j\right)-\bar{T_{k}^{\prime }}\right)}^{2}}\cdot \sqrt{\sum_{i,j}{\left(W\left(u,v,i,j\right)-\bar{W\left(u,v\right)}\right)}^{2}}}$$

where $T_{k}^{\prime }$ is the template at level k, $\bar{T_{k}^{\prime }}$ is its mean intensity, and $\bar{W\left(u,v\right)}$ is the mean intensity of the candidate window in $I_{r,k}$ at position $\left(u,v\right)$.

Since the image pair $\left(I_{l},I_{r}\right)$ has been epipolarly rectified, the search is restricted to a local region. For each level k, the search region is centered on an initial position $\left(u_{0,k},v_{0,k}\right)$ and the position with the maximum score is selected as the optimal location:

(2) $$\left(u_{k}^{*},v_{k}^{*}\right)=\underset{\begin{array}{c} \left|u_{k}-u_{0,k}\right|\leq d_{u}\\ \left|v_{k}-v_{0,k}\right|\leq d_{v} \end{array}}{\mathrm{arg max}}ZNCC\left(u_{k},v_{k}\right)$$

where $\left(u_{k}^{*},v_{k}^{*}\right)$ denote the optimal coordinates at level k, and $d_{u}$ and $d_{v}$ define the horizontal and vertical search ranges, respectively. At the initial level $L_{N}$, the horizontal search is performed globally. Then, the optimal position $\left(u_{k}^{*},v_{k}^{*}\right)$ is scaled by a factor s (typically s=2) to serve as the initial position for the next finer level $\left(k-1\right)$, according to the relation $\left(u_{0,k-1},v_{0,k-1}\right)=\left(s\cdot u_{k}^{*},s\cdot v_{k}^{*}\right)$.

**Figure 3:**
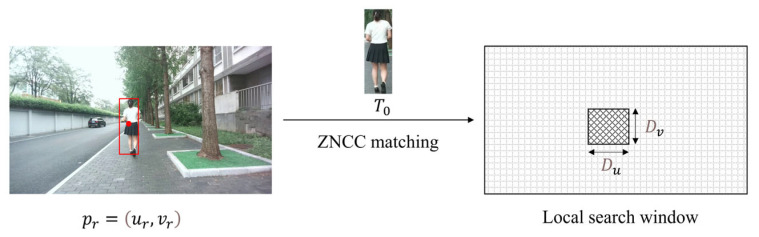
Schematic of the second-stage matching process. The original template $T_{0}$ is used to refine the matching in the original image $I_{r}$ within a local search window, which is defined by a width of $D_{u}$ and a height of $D_{v}$.

Subsequently, in the second-stage matching, we define a local search window at the finest level $L_{0}$, which corresponds to the original image $I_{r}$. The rectangular region $\Omega_{LSW}$ centered at the first-stage matching point $p_{r}=\left(u_{r},v_{r}\right)$. $\Omega_{LSW}$ is defined as:

(3) $$\Omega_{LSW}=\left\{\left(u,v\right)\;|\;\left|u-u_{r}\right|\leq D_{u},\;\left|v-v_{r}\right|\leq D_{v}\right\}$$

where $D_{u}$ and $D_{v}$ determine the size of $\Omega_{LSW}$. Within $\Omega_{LSW}$, the position yielding the maximum score against the original template $T_{o}$ is taken as the final match $p_{r}=\left({\hat{u}}_{r},{\hat{v}}_{r}\right)$, as shown in Figure 3.

### 2.3. Three-View Constraint Optimization

Using the matching pixel coordinates $p_{l}=\left(u_{l},v_{l}\right)$ in image $I_{l}$ and $p_{r}=\left({\hat{u}}_{r},{\hat{v}}_{r}\right)$ in image $I_{r}$, we first compute an initial point to establish a depth prior. The depth estimate is refined using a similarity measure constrained by all three views. This process consists of two main parts: (1) initial point triangulation and search space construction, and (2) maximization of the three-view consistency along the defined search depth axis.

#### 2.3.1. Initial Point Triangulation and Search Space Construction

The initial point can be computed from a pair of matching pixel coordinates using the reprojection matrix $Q_{lr}$, in the left camera coordinate system. Given $p_{l}=\left(u_{l},v_{l}\right)$, $p_{r}=\left({\hat{u}}_{r},{\hat{v}}_{r}\right)$, and the disparity $d=u_{l}-{\hat{u}}_{r}$, the initial point $P_{0}={\left(X_{0},Y_{0},Z_{0}\right)}^{T}$ can be calculated as:

(4) $$P_{0}=[X_{0},Y_{0},Z_{0},1{]}^{T}=\frac{1}{\omega }Q_{lr}[u_{l},v_{l},d,1{]}^{T}$$

where ω is the normalization coefficient for the homogeneous coordinates.

The search space is then reduced to a one-dimensional depth axis, whose direction is defined as the unit vector pointing from the left camera’s optical center $O_{l}$ to the initial point $P_{0}$. The unit vector α is given by:

(5) $$\alpha =\frac{P_{0}-O_{l}}{\left\|P_{0}-O_{l}\right\|}\in R^{3}$$

The search extent along this axis (Δz) is determined by disparity uncertainty. We use the heuristic that objects appearing larger in the image may possess greater depth uncertainty. Therefore, we associate the disparity uncertainty δd with $w_{0}$, the width of the initial ROI, such that $\delta d=\lambda_{z}\cdot w_{0}$. Here, $\lambda_{z}$ is a scaling factor that controls the search range. The relationship between this disparity uncertainty δd and the depth search range Δz is given by the approximation $\Delta z\approx \frac{f\cdot B_{lr}}{d^{2}}\delta d$, where f is the focal length of the left camera and $B_{lr}$ is the baseline distance (can be derived from $Q_{lr}$), and d is the initial disparity estimate of the target.

For the candidate depth $z\in \left[-\Delta z,\Delta z\right]$ along the search axis, the corresponding candidate point $P_{z}$ can be calculated as:

(6) $$P_{z}=P_{0}+z\cdot \alpha$$

#### 2.3.2. Depth Optimization

Within the defined depth search segment, we sample a set of candidate 3D points $\left\{P_{z}\right\}$ with a uniform step size, denoted as $z_{step}$. Each candidate point $P_{z}$ is then reprojected onto the left, right, and top image planes to obtain the corresponding points, respectively. The projected points, $p_{lz}$, $p_{rz}$, and $p_{tz}$, are given by

(7) $$p_{lz}=\pi_{l}^{\prime }\left(P_{z}\right)$$

(8) $$p_{rz}=\pi_{r}^{\prime }\left({R_{lr}^{\prime }P}_{z}+t_{lr}^{\prime }\right)$$

(9) $$p_{tz}=\pi_{t}\left(R_{lt}\cdot \left(R_{rect\_l}^{-1}P_{z}\right)+t_{lt}\right)$$

Here, $\pi_{l}^{\prime }$ and $\pi_{r}^{\prime }$ represent the projective transformations for the rectified left and right cameras, while $\pi_{t}$ is the projective transformations for the top camera. The term $R_{rect\_l}^{-1}$ represents the rotation matrix that transforms from the rectified left camera’s coordinate system back to the original left camera’s coordinate system.

**Figure 4:**
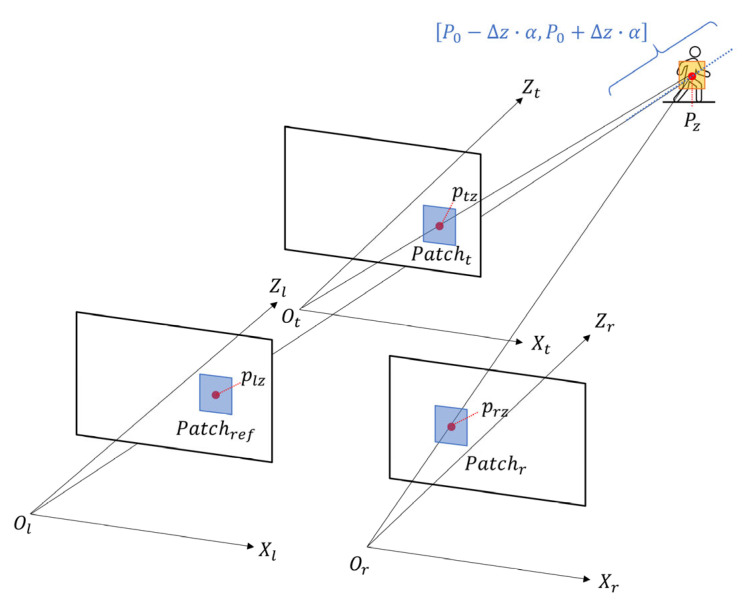
Schematic of the three-view constraint optimization. A candidate point $P_{z}$ is projected onto three image planes. Then, matching patches ($Patch_{ref},$ $Patch_{r}$, $Patch_{t}$) are extracted, centered on projected points ($p_{lz}$, $p_{rz}$, $p_{tz}$), to evaluate the similarity scores across the views.

The total similarity function is defined by the photometric consistency of image patches between the different views, as shown in Figure 4. Specifically, it aggregates the patch similarity scores from the $\left(I_{l},I_{r}\right)$ and $\left(I_{l},I_{t}\right)$ image pairs. The maximum score corresponds to the most refined depth estimation, as it indicates the highest degree of similarity among the corresponding patches in all three views.

The process of depth optimization involves the following steps:

- The reference patch, $Patch_{ref}$, of size M∗M pixels, is extracted from the left view $I_{l}$, centered at the point $p_{l}=\left(u_{l},v_{l}\right)$. The patch size M is determined by the equation $M=r_{m}\cdot min\left(w_{0},h_{0}\right)$, where $w_{0}$ and $h_{0}$ are the width and height of the initial ROI, respectively. The term $r_{m}$ is a scaling factor that relates the patch size to the size of the ROI.
- For each candidate point $P_{z}$, we extract corresponding patches, $Patch_{r}$ and $Patch_{t}$, from the right view $I_{r}$ and the top view $I_{t}$, centered on their respective projected coordinates, $p_{rz}$ and $p_{tz}$.
- The similarity scores are then calculated between $Patch_{ref}$ and the patches from the right and top views ($Patch_{r}$ and $Patch_{t}$, defined as:
  (10) $$ZNCC_{lr}=ZNCC\left(Patch_{ref},Patch_{r}\right)$$
  (11) $$ZNCC_{lt}=ZNCC\left(Patch_{ref},Patch_{t}\right)$$
- The total similarity score for a candidate point $P_{z}$ is defined as the weighted sum of the individual scores:
  (12) $$C\left(z\right)=\lambda_{r}\cdot ZNCC_{lr}+\lambda_{t}\cdot ZNCC_{lt},\;\;\;\;\;\;\lambda_{r}+\lambda_{t}=1$$
  where $\lambda_{r}$ and $\lambda_{t}$ are the weighting coefficients for the right and top views, respectively.
- By iterating through all candidate points $\left\{P_{z}\right\}$, the point that yields the maximum total score is selected as the optimal estimate, $P^{*}=\arg\underset{z^{*}}{\max}C\left(z\right)$.

## 3. Experimental Results

To evaluate the performance of the method proposed in this paper, we constructed a real-world trinocular stereo vision dataset captured from two sidewalk scenes. The dataset we constructed contains 12 sequences of pedestrians moving within a range of 2 to 18 m, totaling 3943 valid frames.

### 3.1. Dataset Collection and Parameters

#### 3.1.1. Dataset Collection

**Figure 5:**
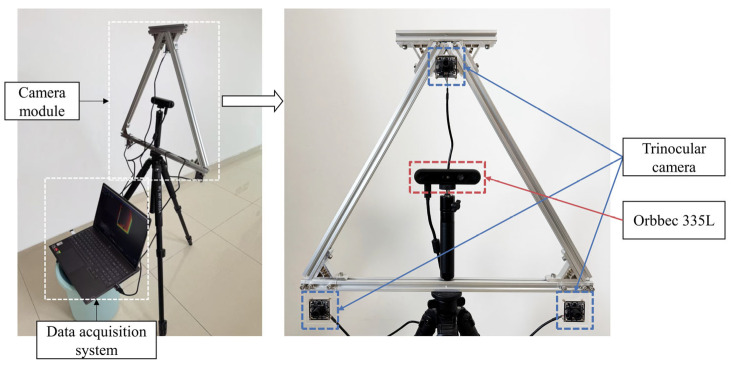
The experimental platform, which includes a camera module and a laptop for data acquisition. The camera module consists of our proposed trinocular vision system and a commercial depth camera (Orbbec-335L).

As shown in Figure 5, the experimental platform integrates our trinocular vision system with a commercial depth camera (Orbbec-335L, Orbbec Inc., Shenzhen, China) and the three cameras are arranged in an approximately equilateral triangle configuration with a baseline length of about 50 cm. The trinocular system consists of three IMX415 (Sony, Beijing, China) camera modules connected to a computing platform via USB 2.0 interfaces. We implemented synchronized image acquisition using Python 3.9. All images were captured at a resolution of 1280 × 720 pixels and a frame rate of 30 FPS.

**Figure 6:**
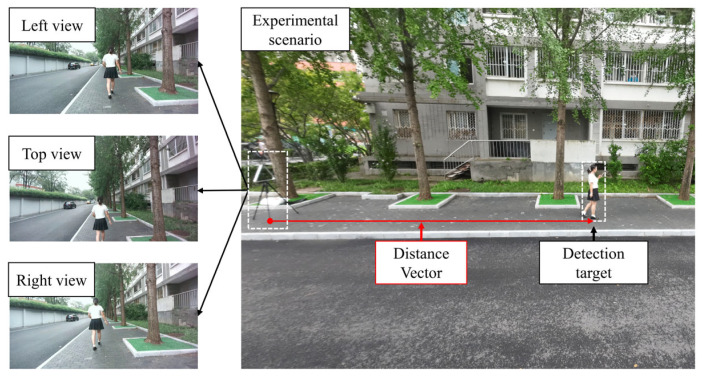
An illustration of the experimental setting. The left, right, and top views are captured by the trinocular system, and an additional reference camera is used to calculate the ground truth distance to the detected target.

**Figure 7:**
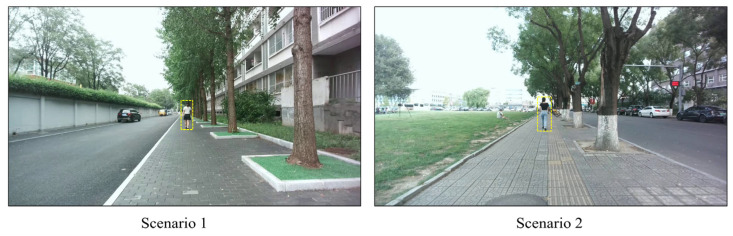
Sample frames in the left camera view that illustrate the two sidewalk scenes (Scenario 1 and Scenario 2) for data collection.

The dataset we constructed contains 12 sequences of pedestrians moving within a range of 2 to 18 m, captured from two sidewalk scenes. The primary scene includes 8 sequences of moving pedestrians, totaling 2956 valid frames. To facilitate cross-scene validation, a supplementary scene was captured, contributing an additional 4 sequences, totaling 987 valid frames, and featuring different lighting conditions and backgrounds. To capture the ground truth for localization, an additional reference camera was positioned to overlook the entire measurement area. The precise world coordinates of the target pedestrian were acquired by post-processing the video from this camera. Specifically, this approach leverages a homography transformation to map annotated 2D pixel coordinates from the static reference camera’s image plane to the 2D world ground plane, which is a standard methodology for such evaluations [49]. These coordinates were used to calculate the true distance between the target and the trinocular system (Figure 6). Figure 7 shows sample frames in the left camera view that illustrate the two sidewalk scenes for data collection.

#### 3.1.2. Parameters

We compare the method proposed in this paper with two binocular baseline methods. First, we adopt the SGBM-based positioning approach [30], utilizing the left and right camera views from our trinocular module as input. This allows us to compare the performance improvement of a trinocular system over a binocular one under an identical baseline of 50 cm. SGBM is a classic and widely used binocular method, which we implemented in OpenCV. The key parameter configurations for the SGBM algorithm and our proposed method, which contains no learnable parameters, are provided in Appendix B.1 and Appendix B.2, respectively. For our second comparison, we selected the Orbbec-335L, a commercial depth camera with a baseline of 9.5 cm, to provide a practical comparison against our wide-baseline system. For our third comparison, we adopt RAFT-Stereo [20], a widely adopted deep learning-based stereo model, utilizing the left and right camera views from our trinocular module as input. For this, we utilize two officially pre-trained models: the RAFT-Stereo Middlebury model, which we refer to as the standard model, and the Realtime model, both of which were applied directly without any fine-tuning on our dataset to ensure a fair assessment of their generalization capabilities. For the real-time version, we use the configuration with the number of down-sampling stages set to 3, the number of GRU layers to 2, and iterative refinements to 7.

The cameras of the trinocular system are calibrated using the checkerboard calibration method available in the MATLAB 2025a Toolbox, following the procedure described in Section 2.1. The calibrated intrinsic and extrinsic parameters (relative to the left camera coordinate system) are presented in Table 1 and Table 2.

### 3.2. Accuracy Evaluation

#### 3.2.1. Comparison of Accuracy

For accuracy evaluation, we compare our proposed method (denoted as Match + Trino) with four baseline methods: the SGBM algorithm, the Orbbec stereo camera, RAFT-Stereo, and RAFT-Stereo (Realtime). To ensure a fair comparison, all methods are evaluated under a consistent framework. Specifically, the initial Regions of Interest (ROIs) for our method, SGBM, and both RAFT-Stereo versions are generated by a single detector operating on the left camera view. For the Orbbec stereo camera, the ROI is extracted from its own RGB image. For these baseline methods, the target’s depth is then determined by extracting the closest valid depth value near the center of its ROI from their respective disparity or depth maps.

**Figure 8:**
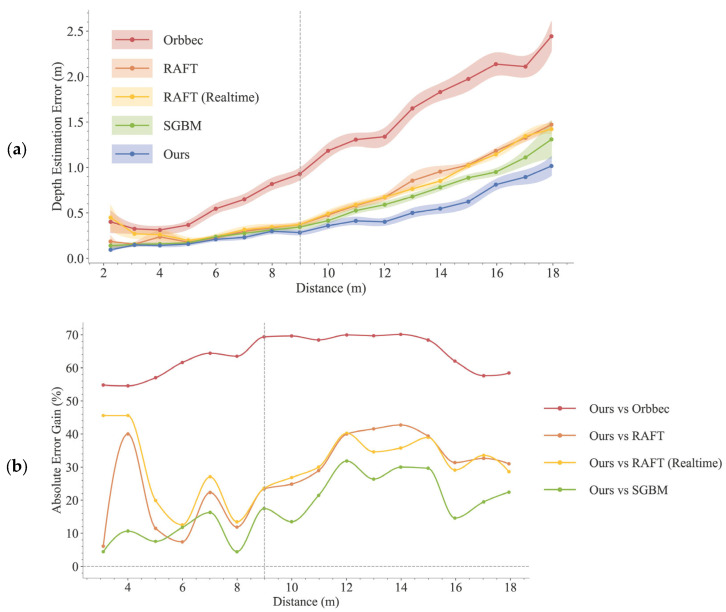
Quantitative comparison of localization accuracy with 95% confidence intervals (shown as shaded areas). Data are aggregated into 1-meter-wide distance bins centered at values from 2 m to 18 m, with sample counts typically around 240 per bin. (**a**) The mean absolute error of our method (Match+Trino), SGBM, the Orbbec camera, RAFT-Stereo, and RAFT-Stereo (Realtime). (**b**) The relative performance gain of our method over the SGBM, the Orbbec camera, RAFT-Stereo, and RAFT-Stereo (Realtime).

The quantitative evaluation of localization accuracy is presented in Figure 8. Figure 8a illustrates the mean absolute error of the five methods, and Figure 8b quantifies the performance gain of our method relative to the four baselines.

As shown in Figure 8a, the localization error for all methods tends to increase with distance. Compared to other binocular baseline methods, our proposed method consistently demonstrates a lower error across the evaluated range (2 to 18 m). The performance gap is particularly evident at longer ranges. For instance, at approximately 18 m, our method’s depth estimation error is 1.02 m, substantially lower than that of SGBM (1.31 m), RAFT-Stereo (Realtime) (1.42m), RAFT-Stereo (1.47 m), and the Orbbec camera (2.44 m).

Figure 8b quantifies this improvement by showing the relative gain of our method over the other methods, representing the percentage reduction in error achieved by our approach. Beyond the 9 m threshold (indicated by the gray dashed line), our method reduces the depth estimation error by over 54% compared to Orbbec and typically by 20% to 40% compared to SGBM, RAFT-Stereo, and RAFT-Stereo (Realtime).

#### 3.2.2. Ablation Study

**Figure 9:**
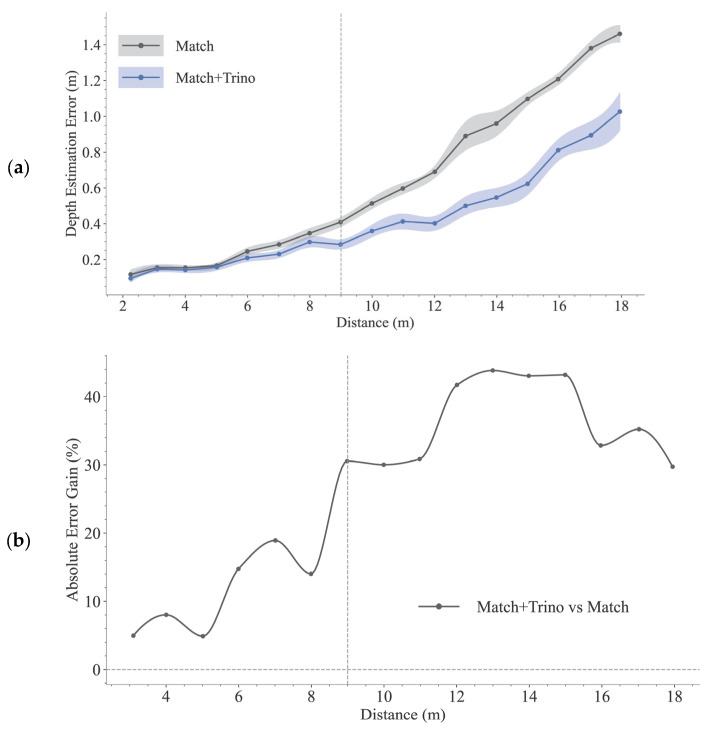
Quantitative results of the ablation study with 95% confidence intervals (shown as shaded areas). Data are aggregated into 1-meter-wide distance bins centered at values from 2 m to 18 m, with sample counts typically around 240 per bin. (**a**) The mean absolute error of the initial (Match) and final (Match + Trino) results. (**b**) The relative performance gain of the final results over the initial results.

To further validate the effectiveness of the three-view constraint optimization module proposed in Section 2.3, we conducted an ablation study. We compare the initial results obtained from multi-scale template matching (denoted as Match) with the final results after optimization (denoted as Match + Trino). The quantitative results of the ablation study are presented in Figure 9.

As shown in Figure 9a, the error after three-view optimization (Match + Trino) is consistently lower than the error of the initial results (Match) across the entire distance range. This result shows that introducing the third viewpoint as a geometric constraint effectively corrects the initial depth estimate and is important to enhancing the accuracy of our method. The gain curve in Figure 9b further quantifies this trend, indicating that the optimization yields progressively more benefit at longer distances. Beyond the 9 m threshold (indicated by the gray dashed line), the optimization consistently reduces the error by 30% to 44%, underscoring the Trino module’s critical role in long-range accuracy.

Table 3 provides a comprehensive performance comparison of each method on the entire dataset, summarizing key error statistics including mean absolute error (MAE), root mean square error (RMSE), and standard deviation (STD).

As shown in Table 3, the results show our method achieves an MAE of 0.435 m, an RMSE of 0.615 m, and an STD of 0.434 m on the dataset. The statistical error values are lower than other methods, indicating that our approach has better consistency and stability.

Furthermore, we evaluate our method’s accuracy under different parameter settings. Specifically, we vary four parameter groups relative to the default configuration defined in Appendix B.2: (i) the view weights $\lambda_{r}$ and $\lambda_{t}$, (ii) the patch size ratio $r_{m}$, (iii) the depth step size $z_{step}$, and (iv) the depth range scale $\lambda_{z}$. The following tables report the MAE across three distance bins (2–6 m, 6–12 m, and 12–18 m), with 95% confidence intervals. The sample counts for each bin are 744, 1619, and 1550, respectively.

The results in Table 4 show that reducing the top view weight $\left(\lambda_{r}=0.75,\lambda_{t}=0.25\right)$ increases the error in all distance bins. This confirms the particular importance of the vertical geometric constraints provided by the top view, especially at longer distances. Conversely, increasing the top-view weight $\left(\lambda_{r}=0.25,\lambda_{t}=0.75\right)$ beyond the default configuration $\left(\lambda_{r}=0.5,\lambda_{t}=0.5\right)$ yields comparable performance with no significant improvement.

Table 5 demonstrates that patch size is a key factor affecting accuracy. Reducing the patch radius ($r_{m}$) leads to a clear increase in MAE, with long-distance errors rising from 0.7 m to 1.17 m. This indicates that excessively small patches fail to capture sufficient texture information from the target, resulting in less reliable similarity estimations. Conversely, increasing $r_{m}$ to 0.7 achieves performance comparable to the default setting of 0.5.

The results in Table 6 and Table 7 show the impact of the depth search step size ($z_{step}$) and the depth range scale ($\lambda_{z}$). The results for different configurations are consistent, confirming that the multi-scale template matching stage provides effective initial depth estimates. This allows the subsequent depth optimization to refine them without requiring an excessively large search range. Therefore, the default settings of $z_{step}=0.1$ m and $\lambda_{z}=0.15$ balance the computational overhead of the optimization phase while maintaining accuracy.

### 3.3. Computational Performance

#### 3.3.1. Comparison of Computational Efficiency

We evaluated the computational efficiency of the algorithms on 1280 × 720 resolution images using an experimental platform equipped with an Intel Core i7-14700KF CPU and an NVIDIA GeForce RTX 4090 GPU. The evaluation included our proposed method (Match + Trino) and its individual components, as well as three baseline algorithms: SGBM, RAFT-Stereo, and RAFT-Stereo (Realtime). Table 8 presents the average run time and resulting throughput for each.

As shown in Table 8, our method exhibits high computational efficiency for localizing individual targets. Unlike baseline methods with a fixed per-frame cost, our method scales linearly with the number of targets. For a single target (N=1), our complete pipeline requires about 3.13 ms, achieving a throughput of approximately 319 FPS, which exceeds the performance of RAFT-Stereo (Realtime) (47.0 FPS), SGBM (19.6 FPS), and the standard RAFT-Stereo (1.35 FPS). Separately, the Match step requires about 0.98 ms and the Trino optimization step takes approximately 2.15 ms, showing that the Trino optimization is the more computationally intensive component in our method.

Furthermore, the relationship between algorithm performance and target size is illustrated in Table 9, where target size is measured by the pixel area of the target’s ROI.

As shown in Table 9, the runtimes of SGBM, RAFT-Stereo, and RAFT-Stereo (Realtime) are all nearly independent of the target size, as they perform dense computations across the entire image. In contrast, the computational cost of our proposed method trends upward as the target’s size decreases (i.e., as the target becomes more distant). It is most efficient for medium-sized targets (2.24 ms for the 128^2^ px bin) and increases for smaller, more distant targets.

#### 3.3.2. Time Breakdown

**Figure 10:**
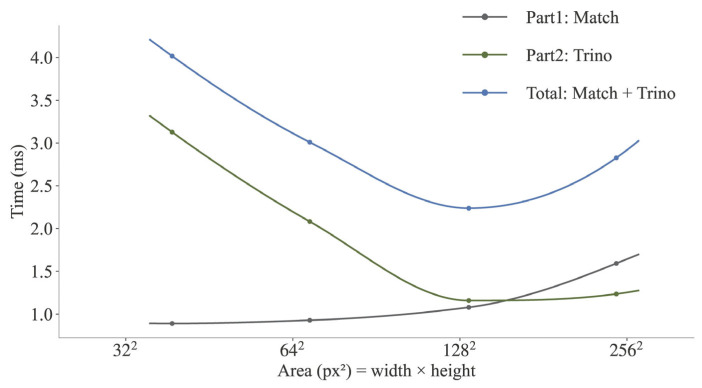
Impact of target size on the runtimes of the proposed method (Match + Trino) and its individual components (Match and Trino). Data are categorized into the same pixel area bins as in Table 9 (e.g., 32^2^, 64^2^, 128^2^), which also reports the mean area and sample counts for each bin.

Figure 10 shows the runtime trends for the total algorithm and its two main components (Match and Trino) as a function of the target’s pixel area. Results in Figure 10 are binned by theoretical centers (e.g., 32^2^, 64^2^, 128^2^). Points are plotted at the empirical mean of each bin.

As shown in Figure 10, the runtime of the Match stage remains relatively stable at approximately 1.5 ms and is less affected by the target’s size. In contrast, the Trino step is the primary factor influencing the method’s total runtime, causing it to increase as the target’s size decreases. Thus, the total runtime reflects the combined contributions of these two components. For smaller targets (approaching the 1024 px^2^ bin), the total runtime reaches approximately 4.0 ms.

Furthermore, Table 10 presents a breakdown of the computational cost for the Match and Trino stages, including the average patch size M, depth search range Δz, GFLOPs, and latency for each target size bin. For the Match stage, GFLOPs are computed as the cumulative cost of all ZNCC evaluations over the multi-scale search and refinement. The cost of a single ZNCC on a w×h template is approximated as 10 wh FLOPs. For the Trino stage, FLOPs are estimated as $N_{z}\cdot \left(2\cdot 10\cdot M^{2}+\kappa \right)$, where $N_{z}=\left\lfloor \frac{2\cdot \Delta z}{z_{step}}\right\rfloor \;+1$ is the number of depth samples, M is the patch size, and the factor of 2 accounts for two ZNCC evaluations per sample. The term κ denotes a fixed non-pixel-level overhead per depth sample, which was calibrated from the measured latencies using a simple linear model to cover costs from the Python loop, camera projections, and patch extraction.

The results in Table 10 show performance characteristics for the two stages. For the Match stage, the required computation (GFLOPs) and the resulting latency increase with target area. In contrast, the Trino stage shows higher latency, indicating a lower effective throughput of approximately 1.9–2.3 GFLOPs/s, compared to the Match stage’s median effective throughput of about 210 GFLOPs/s. This gap arises because the Match stage benefits from a highly optimized sliding-window kernel, while the Trino stage is limited by per-depth sample overheads and less efficient memory access.

To complete the pedestrian localization pipeline, we integrated our method with the YOLOv8-s object detector [50] for initial ROI generation. On our experimental dataset, the end-to-end system achieved an average inference time of 7.52 ms per frame for a single pedestrian, which consists of 4.39 ms for the YOLOv8-s detector and 3.13 ms for our proposed trinocular localization algorithm. The detector was configured with the pretrained yolov8s.pt weights. During testing, the input image size was set to 1280, the confidence threshold to 0.5, and the intersection over union (IoU) threshold for non-maximum suppression was 0.70.

To evaluate the system’s viability for real-time deployment with multiple pedestrians, we consider that the YOLOv8-s detector processes the entire image once, while our localization algorithm is applied sequentially to each of the N detected pedestrians. Consequently, the total processing time per frame can be estimated as $\left(4.39+N\times 3.13\right)$ ms. This indicates that for a standard 30 Hz video stream (which provides a 33.3 ms budget per frame), our system can maintain real-time performance for up to N=9 pedestrians, with a total processing time of approximately 32.56 ms.

### 3.4. Qualitative Analysis of Matching and Detection Inaccuracies

**Figure 11:**
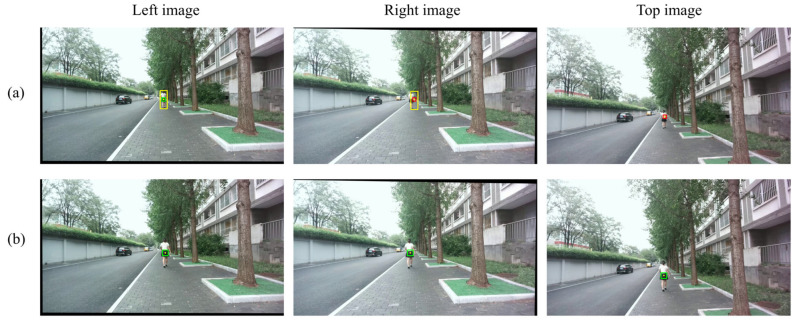
Correction of a simulated matching inaccuracy by the Trino stage. A 15-pixel rightward offset was manually added to the initial disparity from the Match stage. (**a**) The erroneous 3D point projected back onto the three views, with extracted patches highlighted in red and bounding boxes outlined in yellow. (**b**) After applying the Trino stage, the corresponding patches (highlighted in green) are correctly aligned with the target.

**Figure 12:**
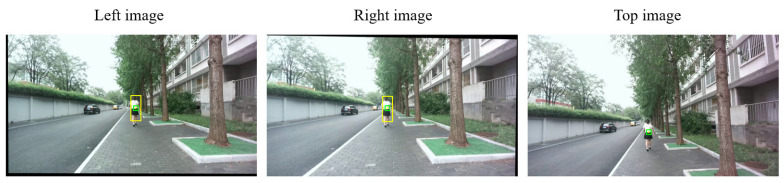
Correction of a simulated detection inaccuracy by the Trino stage. The initial ROI in the left view (yellow box) was manually shifted upward and to the right of the target, resulting in an offset correspondence in the right view. After applying the Trino stage, the projected patches (highlighted in green) are correctly located on the pedestrian, demonstrating tolerance to small initial detection errors.

We present two qualitative examples illustrating matching and detection inaccuracies. In these cases, we manually introduced offsets to simulate errors in both the matching stage (Figure 11) and the initial ROI (Figure 12).

Figure 11 demonstrates the capability of the Trino stage to correct inaccurate input depth computed by the Match stage. To simulate a matching inaccuracy, we manually added a 15-pixel rightward offset to the initial disparity obtained from the Match stage. As shown in Figure 11a, the matched bounding box in the right view is shifted relative to the input detection in the left view (both boxes are shown in yellow). The initial 3D point corresponding to this incorrect match is then projected back onto the three views, with the extracted patches highlighted in red. Figure 11b shows the result after applying the three-view optimization in the Trino stage. The corresponding patches (highlighted in green) appear in the correct positions on the target, indicating that the matching inaccuracy has been corrected.

Figure 12 presents a simulated detection inaccuracy, where the initial ROI in the left view (shown as a yellow box) is slightly offset upward and to the right of the target. Consequently, the matching result yields a similarly offset correspondence in the right view (shown as a yellow box), since it relies on the inaccurate ROI in the left view as the template. After applying the Trino stage, the resulting 3D point is projected onto the three views. The corresponding patches are highlighted in green, which are correctly located on the target pedestrian. Since our optimization extracts patches from the ROI center, it is tolerant to small offsets in the initial detection from the left view.

## 4. Discussion

Experimental results show that the method proposed in this paper performs better in accuracy and computational efficiency. The comparison with the SGBM and RAFT-Stereo methods was conducted under an identical baseline, as their input was sourced from the left and right cameras of our trinocular module. This configuration provides a direct validation of the performance advantage of trinocular over binocular systems.

In terms of positioning accuracy, our method achieves a lower error than the binocular methods (SGBM, the RAFT-Stereo methods, and the depth camera). The main reason is that our method is target-oriented, focusing computational resources on the target ROI. In contrast, the SGBM algorithm is a dense matching algorithm, which is prone to generating errors in low-texture areas, such as a pedestrian’s clothing. Similarly, the RAFT-Stereo methods, despite being state-of-the-art deep learning approaches, also exhibit higher errors. This is primarily because we utilized models pre-trained on standard datasets without any fine-tuning on our specific data. Consequently, the features learned by the pre-trained models do not generalize well to our dataset, as the gap between the training data and outdoor pedestrian scenes leads to degraded performance. The depth camera’s performance also degrades outdoors, particularly at long distances.

Furthermore, the experimental results indicate that the accuracy advantage of our method increases with distance, which can be attributed to the additional geometric constraint introduced by the third viewpoint from the top camera. In traditional binocular stereo vision, the horizontal disparity of a given point decreases as the distance increases. At long distances, the binocular system becomes more sensitive to disparity errors, causing small pixel estimation errors to be amplified into significant depth errors.

Our trinocular system introduces a vertical viewpoint, providing a non-collinear geometric constraint that complements horizontal disparity. The observation from the top camera effectively constrains the target’s position along the depth axis. This miti-gates the ambiguity inherent in the pixel estimation of horizontal stereo systems at long ranges. This additional constraint addresses a key limitation of binocular systems, enabling precise long-range measurements. The results of the ablation study (Table 4 and Figure 9) also support the critical role of the third camera.

In terms of computational efficiency, our method outperforms both the classic SGBM algorithm and RAFT-Stereo (Realtime) in runtime. The standard RAFT-Stereo, requiring 739.43 ms per frame, is ill-suited for real-time applications. As detailed in Table 8, our complete localization process handles a single target in an average of 3.13 ms. This advantage stems from the fact that the runtime of dense-matching methods, such as SGBM and RAFT-Stereo, is strongly coupled with image resolution, resulting in a significant computational load for our high-resolution (1280 × 720) dataset.

In contrast, our method mitigates this dependency. First, the initial matching process efficiently narrows the search space via a multi-scale strategy. Subsequently, the computational cost of the depth optimization under the three-view constraint is determined not by image resolution, but by the depth search range. When the multi-scale template matching stage provides a relatively accurate initial depth estimate, the subsequent optimization can refine it without requiring an excessively large search range. This targeted approach remains more efficient than the full-image computation required by both the SGBM and RAFT-Stereo methods.

The methodology proposed in this study demonstrates its potential for accurate localization in outdoor environments. It is suitable for applications such as autonomous driving, robot navigation, and intelligent surveillance. Moreover, the trinocular system can be further customized and optimized by adjusting physical deployment configuration, ranging from deployments on urban transport infrastructure and buildings to lightweight wearable devices. Nevertheless, several limitations remain, offering valuable directions for future research.

The proposed method operates as a localization-after-detection pipeline, which makes its performance fundamentally dependent on the success and accuracy of the initial 2D object detector. This dependency introduces a potential point of failure: a missed detection interrupts localization entirely, while an imprecise bounding box propagates errors and degrades the final estimate. To address this, future work should explore a tighter and more synergistic integration of the detection and localization modules. For example, an end-to-end network that jointly optimizes both tasks could reduce reliance on the accuracy of the initial bounding box. Alternatively, embedding the localization algorithm within a tracking framework would allow position estimates from previous frames to inform and refine object detection in subsequent frames, thereby creating a more robust and resilient system.

Furthermore, the choice of similarity measure method is largely determined by practical hardware deployment and application scenarios. In this work, we employed ZNCC as the similarity metric. While ZNCC is effective and efficient, its performance is constrained in challenging scenarios. The reliability of its matching score degrades significantly under occlusions, severe non-linear illumination changes, or when targets exhibit low-texture surfaces.

To extend the applicability of our framework, particularly for safety-critical systems, future research should explore more advanced matching strategies. Deep learning-based descriptor heads pre-trained on large-scale datasets are promising alternatives, as they excel at handling complex appearance variations and have demonstrated superior performance in challenging conditions. However, their adoption requires a careful consideration of the trade-off between their enhanced robustness and significant computational cost, especially for real-time applications. It is also important to explicitly address the occlusion challenge in crowded environments. Promising approaches include part-based matching, which reasons about visible object components, and robust aggregation methods like sub-block voting, which build consensus from partially matched regions. With these further improvements, we believe the proposed trinocular framework can be developed into a widely applicable solution across diverse application scenarios.

## 5. Conclusions

This paper presents a trinocular stereo vision system for pedestrian localization, with cameras arranged at the vertices of an equilateral triangle and parallel optical axes. Experimental results on our custom dataset show that our method achieves high accuracy, with a mean absolute error of 0.43 m, and high computational efficiency with an average processing time of 3.13 ms per target. This performance exceeds that of the binocular SGBM algorithm (0.536 m and 19.6 FPS), and RAFT-Stereo (Realtime) (0.621 m and 47.0 FPS), and the standard RAFT-Stereo (0.623 m and 1.35 FPS). Empirical results show that the performance advantage becomes more pronounced at longer ranges. For distances beyond 9 m, our method achieves a relative error reduction of 20% to 40% compared to the binocular methods using equivalent baseline settings. When integrated with a YOLOv8-s object detector, the end-to-end system is capable of maintaining real-time performance (>30 Hz) for up to nine pedestrians, with a total processing time of approximately 32.56 ms, underscoring its practical deployment viability.

## Figures and Tables

**Table 1:** Intrinsic matrices of the trinocular camera system.

Camera	$f_{x}$	$f_{y}$	$c_{x}$	$c_{y}$
$K_{l}$	711.9746	710.7513	656.8128	370.2462
$K_{r}$	709.7325	708.6549	639.3270	338.5957
$K_{t}$	720.5992	721.1380	626.4810	372.2269

**Table 2:** Extrinsic parameters (Relative to the left camera coordinate system).

Pair	Rotation R	Translation t (mm)
$\left(R_{lr},t_{lr}\right)$	$\left[\begin{array}{ccc} 0.9995 & 0.0174 & 0.0280\\ -0.0176 & 0.9998 & 0.0035\\ -0.0280 & -0.0040 & 0.9996 \end{array}\right]$	$\left[\begin{array}{c} -468.9070\\ 5.3564\\ 4.5990 \end{array}\right]$
$\left(R_{lt},t_{lt}\right)$	$\left[\begin{array}{ccc} 0.9999 & 0.0117 & 0.0106\\ -0.0125 & 0.9965 & 0.0831\\ -0.0096 & -0.0833 & 0.9965 \end{array}\right]$	$\left[\begin{array}{c} -226.8953\\ 439.3381\\ -29.7137 \end{array}\right]$

**Table 3:** Comparison of localization errors on the dataset.

Method	MAE (m)	RMSE (m)	STD (m)
Orbbec 335L	1.221	1.549	0.953
SGBM	0.536	0.734	0.501
RAFT-Stereo	0.623	0.800	0.501
RAFT-Stereo (Realtime)	0.621	0.781	0.473
Match (Ours, w/o Opt.)	0.638	0.815	0.508
Match + Trino (Ours)	0.435	0.615	0.434

**Table 4:** Results on the impact of view weights $\left(\lambda_{r}{\;\lambda }_{t}\right)$ on MAE (m) *.

Parameter: $\left(\lambda_{r},\lambda_{t}\right)$	MAE (2–6 m)	MAE (6–12 m)	MAE (12–18 m)
(0.25, 0.75)	0.161 ± 0.009	0.309 ± 0.012	0.695 ± 0.026
(0.5, 0.5) (default)	0.154 ± 0.009	0.313 ± 0.013	0.702 ± 0.026
(0.75, 0.25)	0.167 ± 0.011	0.330 ± 0.014	0.735 ± 0.027

* All MAE values are reported as mean ± 95% confidence interval.

**Table 5:** Results on the impact of patch size ratio ($r_{m}$) on MAE (m) *.

Parameter: $r_{m}$	MAE (2–6 m)	MAE (6–12 m)	MAE (12–18 m)
0.3	0.208 ± 0.011	0.521 ± 0.021	1.169 ± 0.045
0.5 (default)	0.154 ± 0.009	0.313 ± 0.013	0.702 ± 0.026
0.7	0.150 ± 0.008	0.290 ± 0.011	0.699 ± 0.023

* All MAE values are reported as mean ± 95% confidence interval.

**Table 6:** Results on the impact of depth search step ($z_{step}$) on MAE (m) *.

Parameter: $z_{step}$	MAE (2–6 m)	MAE (6–12 m)	MAE (12–18 m)
0.05	0.155 ± 0.009	0.315 ± 0.013	0.709 ± 0.026
0.1 (default)	0.154 ± 0.009	0.313 ± 0.013	0.702 ± 0.026
0.2	0.160 ± 0.009	0.314 ± 0.013	0.695 ± 0.026

* All MAE values are reported as mean ± 95% confidence interval.

**Table 7:** Results on impact of depth range scale ($\lambda_{z}$) on MAE (m) *.

Parameter: $\lambda_{z}$	MAE (2–6 m)	MAE (6–12 m)	MAE (12–18 m)
0.1	0.156 ± 0.009	0.311 ± 0.013	0.712 ± 0.029
0.15 (default)	0.154 ± 0.009	0.313 ± 0.013	0.702 ± 0.026
0.2	0.157 ± 0.009	0.312 ± 0.013	0.709 ± 0.026

* All MAE values are reported as mean ± 95% confidence interval.

**Table 8:** Average run time of algorithms/components.

Algorithms/Components	Average Time (ms)	Throughput (FPS) @ N = 1 *
SGBM	51.02	19.60
RAFT-Stereo	739.43	1.35
RAFT-Stereo (Realtime)	21.26	47.04
Part 1: Match (Ours)	0.98	-
Part 2: Trino (Ours)	2.15	-
Total: Match+Trino (Ours)	3.13	319.49

* Throughput for baseline methods is measured per-frame. For our method, throughput is reported per-target (for N = 1), as its runtime scales with the number of targets.

**Table 9:** Average runtime (ms) for different target sizes.

$\mathrm{Bin}\;\mathrm{Center}\;\left(px^{2}\right)$ *	Mean Area (px^2^)	SGBM (ms)	RAFT-Stereo (ms)	RAFT-Stereo (Realtime) (ms)	Ours (ms)
$32^{2}=1024$	1875.85	51.59	739.04	21.30	4.02
$64^{2}=4096$	5342.40	50.87	740.13	21.26	3.01
$128^{2}=16,384$	18,990.66	50.73	738.74	21.20	2.24
$256^{2}=65,536$	62,398.28	50.21	738.53	21.25	2.83

* Bin center refers to the pre-defined center of the area bins (e.g., 32^2^, 64^2^, 128^2^). Mean area reports the actual average area of samples within each bin. The sample counts for each bin are 1164, 1732, 852, and 195, respectively.

**Table 10:** GFLOPs and latency for match and trino stages.

$\mathrm{Bin}\;\mathrm{Center}\;\left(px^{2}\right)$ *	Patch Size M $\left(px\right)$	Δz(m)	Match GFLOPs	Match Latency (ms)	Trino GFLOPs	Trino Latency (ms)
$32^{2}=1024$	13.5	2.71	0.034	0.890	0.0058	3.126
$64^{2}=4096$	22.1	1.82	0.096	0.928	0.0041	2.081
$128^{2}=16,384$	40.9	1.00	0.342	1.079	0.0027	1.158
$256^{2}=65,536$	69.5	0.57	1.12	1.591	0.0023	1.235

* Bin center refers to the pre-defined center of the area bins (e.g., 32^2^, 64^2^, 128^2^). Patch size M and Δz report the actual average values of samples within each bin. The Trino stage GFLOPs calculation uses $z_{step}=$ 0.1 m (according to Appendix B.2), and the fixed overhead κ≈1.037×105 FLOPs/sample.

## Data Availability

The data presented in this study are available on request from the corresponding author due to privacy reasons.

## Appendix A

The overall methodology is summarized in Algorithm A1. The algorithm details the complete computational process, starting from inputting the images $\left(P_{l},P_{r},P_{t}\right)$, the original target template $T_{0}=\left(u_{l},v_{l},w_{0},h_{0}\right)$ and the set of camera module calibration parameters set S, to executing the multi-scale template matching and three-view constraint optimization, and finally returning the target’s refined position $P^{*}$.

**Algorithm A1** The proposed trinocular stereo vision localization algorithm
**Input:** Left image $P_{l}$, right image $P_{r}$, top image $P_{t}$, original target template (from left image) $T_{0}=\left(u_{l},v_{l},w_{0},h_{0}\right)$, camera parameter set $S=\left(K_{l},K_{r},K_{t},R_{lr},t_{lr},R_{lt},t_{lt},Q_{lr}\right)$. **Output:** The target $P^{*}$ (in the left camera coordinate system). 1: $\left(I_{l},I_{r},I_{t}\right)=$ ImageRectification $\left(P_{l},P_{r},P_{t}\right)$ “Obtaining initial matching from $I_{l},I_{r}$” 2: $T^{\prime }$ = Preprocessing $\left(T_{0}\right)$ 3: Build image pyramids for $I_{r}$ and $T^{\prime }$. 4: **for** k from $L_{N}$ **down to** 0 **do** 5: **if** k is $L_{N}$ **then** 6: Initial $v_{0,k}=v_{l}$ 7: Search $(u_{k}^{*},v_{k}^{*})=\underset{\left|v_{k}-v_{0,k}\right|\leq d_{v}}{\mathrm{arg max}}ZNCC(u_{k},v_{k})$ 8: **else** 9: Initial $\left(u_{0,k},v_{0,k}\right)=\;(s\cdot u_{k+1}^{*},s\cdot v_{k+1}^{*})$ 10: Search $(u_{k}^{*},v_{k}^{*})=\underset{\begin{array}{c} \left|u_{k}-u_{0,k}\right|\leq d_{u}\\ \left|v_{k}-v_{0,k}\right|\leq d_{v} \end{array}}{\mathrm{arg max}}ZNCC(u_{k},v_{k})$ 11: **end if** 12: **end for** 13: Use $T_{0}$ for search: 14: $\Omega_{LSW}=\left\{\left(u,v\right)\;|\;\left|u-u_{r}\right|\leq D_{u},\;\left|v-v_{r}\right|\leq D_{v}\;\right\}$ 15: Find the best matching point $p_{r}=\left({\hat{u}}_{r},{\hat{v}}_{r}\right)$ for $p_{l}=\left(u_{l},v_{l}\right)$ “Optimization based on $I_{l},I_{r},I_{t}$” 16: $P_{0}$ = Triangulation $\left(p_{l},p_{r},Q_{lr}\right)$ 17: **for** z **in** $\left[-\Delta z,\Delta z\right]$ **with** $z_{step}$ **do** 18: Candidate points $P_{z}=P_{0}+z\cdot \alpha$ 19: Extract $\left(Patch_{r}\;,Patch_{t}\right)$ corresponding to the projection point: 20: $\left(p_{lz},p_{rz},p_{tz}\right)=$ Projection $\left(P_{z},S\right)$ 21: Calculate $C\left(z\right)=\lambda_{r}\cdot ZNCC_{lr}+\lambda_{t}\cdot ZNCC_{lt}$ 22: **end for** 23: $P^{*}=\arg\underset{z^{*}}{\max}C\left(z\right)$ 24: **return** $P^{*}$

## Appendix B

### Appendix B.1

The detailed parameter configurations for the SGBM algorithm are provided in Table A1, which serves as a baseline for comparison. Specifically, the fundamental search range for disparity values is defined by minDisparity and numDisparities, with the latter set to 256. The matching process operates on a pixel window of blockSize = 5. The penalty coefficients P1 and P2, which control the smoothness constraint, are set to values of 1176 and 4704, respectively. To improve the quality of the resulting disparity map, post-processing is performed using parameters including disp12MaxDiff = 1, uniquenessRatio = 10, speckleWindowSize = 100, and speckleRange = 32 to filter out noise and unreliable matches. The mode is set to MODE_SGBM_3WAY to enable the full-scale dynamic programming approach for more robust results.

**Table A1:** Parameters for the SGBM algorithm in OpenCV.

Parameter	Value
minDisparity	0
numDisparities	256
blockSize	5
P1	1176
P2	4704
disp12MaxDiff	1
uniquenessRatio	10
speckleWindowSize	100
speckleRange	32

### Appendix B.2

The detailed parameter configurations for our proposed method are provided in Table A2. The parameters are organized into two main components: the multi-scale matching (Match) and the trinocular optimization (Trino).

In the initial matching process, the image pyramid level is set to 3. The pyramid is constructed via OpenCV, which applies a fixed 5 × 5 Gaussian kernel for smoothing prior to each down-sampling step. The parameters governing search template handling, including the expansion ratio ($r_{exp}=1.5$), cropping ratio ($r_{cut}=0.5)$, and area thresholds ($A_{min}=4096$, $A_{max}=65,536$), were determined empirically on our custom dataset to balance the retention of sufficient contextual information for small targets with the need for stable matching in large ones. For the pyramid levels, the horizontal and vertical search ranges are 10 and 2. At the finest level $L_{0}$, the local search window is set to 20 and 20. In the depth optimization process, the disparity uncertainty ratio is set to 0.15, with the deep search sampled at a discrete step size of 0.1. The patch size ratio is set to 0.5, and the weight coefficients for the right and top view matching scores are set to 0.5. This provides equal weight to the evidence from both the right and top views.

**Table A2:** Parameters for the proposed method.

Component	Parameter	Value
Match	N	3
	$A_{min}$	4096
	$A_{max}$	65,536
	$r_{exp}$	1.5
	$r_{cut}$	0.5
	$d_{u}$	10
	$d_{v}$	2
	$D_{u}$	20
	$D_{v}$	20
Trino	$\lambda_{z}$	0.15
	$z_{step}$	0.1
	$r_{m}$	0.5
	$\lambda_{r}$	0.5
	$\lambda_{t}$	0.5
